# Push-Out Bond Strength of Prefabricated Fiberglass Posts in Endodontically Treated Teeth After Two Intracanal Cleaning Protocols

**DOI:** 10.7759/cureus.105466

**Published:** 2026-03-18

**Authors:** Jennifer Parra, Geezer Montoya, Herney Garzón, Mónica Escudero

**Affiliations:** 1 Oral Rehabilitation, Universidad del Valle, Cali, COL; 2 Periodontics, Oral Rehabilitation, and Implantology, Universidad del Valle, Cali, COL

**Keywords:** adhesion, dentin, irrigation, post, prefabricated posts, theracem

## Abstract

Introduction

The different agents and intracanal irrigation systems used in endodontically treated teeth can affect the adhesion of prefabricated posts, which are frequently required to rehabilitate endodontically treated teeth.

Objective

To determine the adhesive resistance strength of fiberglass posts in endodontically treated teeth irrigated with NaOCl (sodium hypochlorite), activated both mechanically and ultrasonically.

Methods

Fourteen teeth that received endodontic treatment were defilled, leaving a 4 mm apical seal, and divided into two groups. Group one was irrigated with NaOCL and subjected to ultrasonic activation, and group two was irrigated with NaOCL and subjected to mechanical activation. DT Light-Post^®^ IllusionX-RO^®^ # 1 (RDT Dental, Saint-Égrève, France) fiberglass posts were cemented using TheraCem® (BISCO, Inc., Schaumburg, Illinois, USA) self-adhesive cement. The samples were aged by storing them at a constant temperature (36°C) for 30 days. Four cuts, each 2.5 mm in length, were then made and three root discs of the cervical, middle, and apical zones were obtained and subjected to a push-out test.

Results

For the 21 analyzed samples, group one presented a higher average adhesive strength (13.60 MPa) compared with group two (11.62 MPa).

Conclusions

The ultrasonic activation group reported the highest average strength at maximum load.

## Introduction

For the rehabilitation of endodontically treated teeth that have lost a large amount of their dental structure, fiberglass posts have been used as an alternative to classic metal posts because their modulus of elasticity is similar to that of dentin, which allows better distribution of occlusal stresses along the axis of the root dentin [1-4]. To cement a post, the tooth must first undergo an intracanal cleaning process as part of the endodontic treatment protocol. This procedure increases the permeability of the dentin and improves the penetration of the adhesive monomers between the demineralized collagen fibers, which results in a better-quality hybrid layer [5].

Due to its antimicrobial properties and mechanism of action, sodium hypochlorite or NaOCl has remained one of the most widely used endodontic intracanal irrigation agents [6]. However, various studies have shown that NaOCl does not produce the desired antibacterial effect when used alone, but its effectiveness has been suggested to improve upon manual, mechanical, ultrasonic or laser activation [7,8]. Owing to the anatomical variations in root canals, mechanical and ultrasonic activation have been proposed to help clean certain remote areas of the root canal that are not adequately cleaned by conventional irrigation methods [9].

To guarantee adequate adhesion of a post to root dentin, three aspects must be considered: surface treatment, cementing system selection and cement polymerization, which can be performed via etching and washing (ER) and self-etching (SE) strategies [5]. Dual self-adhesive cements are considered the first choice for the cementation of fiberglass posts since, compared with photoactivated systems, they do not require the use of conventional adhesive systems, allowing the operator to have greater control over humidity and in smaller areas of the canal that are not visible. In addition, dual self-adhesive cements have a longer working time, which allows for better control during post adaptation since the light emitted by the photopolymerizing device does not reach the most apical areas in the canal, which results in poor conversion of the resin monomers and compromises the integrity of the adhesive interface [10,11]. However, there is insufficient evidence to support that the cleaning system affects the adhesive strength of intraradicular posts.

This study presents the results of an investigation of the adhesive resistance of fiberglass posts cemented in endodontically treated teeth after intracanal cleaning with sodium hypochlorite via two different protocols: mechanical activation by the use of a rotary brush-type attachment mounted on a low-speed, Versa Brush™ (Vista Apex, Racine, Wisconsin, US), and ultrasonic activation by the use of a Biosonic S1 scaler (COLTENE Holding AG, Altstätten, Switzerland).

## Materials and methods

An in vitro experimental study was carried out to determine the adhesive resistance of the interfaces of precast fiberglass posts cemented with self-adhesive cement in endodontically treated human premolars that were irrigated with NaOCL using push-out displacement tests, designed to assess the strength and stability of the posts.

The sample size was calculated using G*Power software (ver 3.1.9.7, Heinrich-Heine-Universität Düsseldorf, Düsseldorf, Germany) and Student’s t-test to evaluate the difference in means between the two independent groups [12]. It was determined that 14 sample bodies (seven in each group) were needed for an effect size of 1.78 (with group averages of 18.96 ± 4.36 ± 12 and 18 ± 3.12 MPa for mechanical and ultrasonic activation, respectively) with a power of 0.8 and an error (α) of 0.05. Samples were subsequently taken from the cuts for evaluation.

The data was entered into an Excel template (Microsoft Corp., Redmond, WA, USA). Data analysis was subsequently carried out using STATA IC15 (StataCorp LLC, College Station, TX, US), and the results were plotted using GraphPad Prism 9 (Dotmatics, Boston, Massachusetts, US). The analyses included calculating measures of central tendency, dispersion and the position of maximum debonding resistance. The measurements from each zone were compared using one-way analysis of variance (ANOVA).

The results from the different groups were compared via Student’s t-tests. Then, the normality and homogeneity of variance of the data were compared via the Shapiro-Wilk and Levene tests, respectively. In addition, the data from each disc within the same group were compared via one-way ANOVA to identify associations between the type of irrigant activation and the maximum resistance force in each of the tooth areas.

Fourteen human premolars were acquired by uni- or biradicular extraction (with prior approval from the Institutional Committee for the Review of Human Ethics of the Faculty of Health of the Universidad del Valle, Cali, Columbia) that met the inclusion criteria for which the root canals were performed.

The roots were endodontically prepared by instrumenting to a working length of 1 mm from the radiographic apex with a standard # 35 apical file. All canals were cut by the same operator. The StepBack^®^ technique [13] was used for irrigation with 5% sodium hypochlorite, and the samples were rinsed with saline solution after each file change, dried with paper tips (META BIOMED Co., Ltd., Cheongju-si, South Korea) and sealed with gutta-percha cones (Hygenic Coltene^®^, COLTENE Holding AG, Altstätten, Switzerland) and endodontic cement (MTA Fillapex, Angelus Indústria de Produtos Odontológicos S/A, Brazil). Then, the cervical part of the tooth was temporarily sealed with Teflon and coltosol (COLTENE Holding AG, Altstätten, Switzerland) before the teeth were stored in dark containers at room temperature with 100% humidity.

After storage, the crown of each tooth 2 mm above the amelocemental junction was removed using a high-speed handpiece while cooling with plenty of water. Then, the length of the root canal was verified, and the root canal was unblocked, leaving 4 mm of gutta-percha as the apical seal. The canals were expanded with the shaping milling system indicated for use with the chosen fiberglass post system (Light-Post^®^ IllusionX-RO^®^ # 1 (RDT Dental, Saint-Égrève, France)).

**Figure 1:**
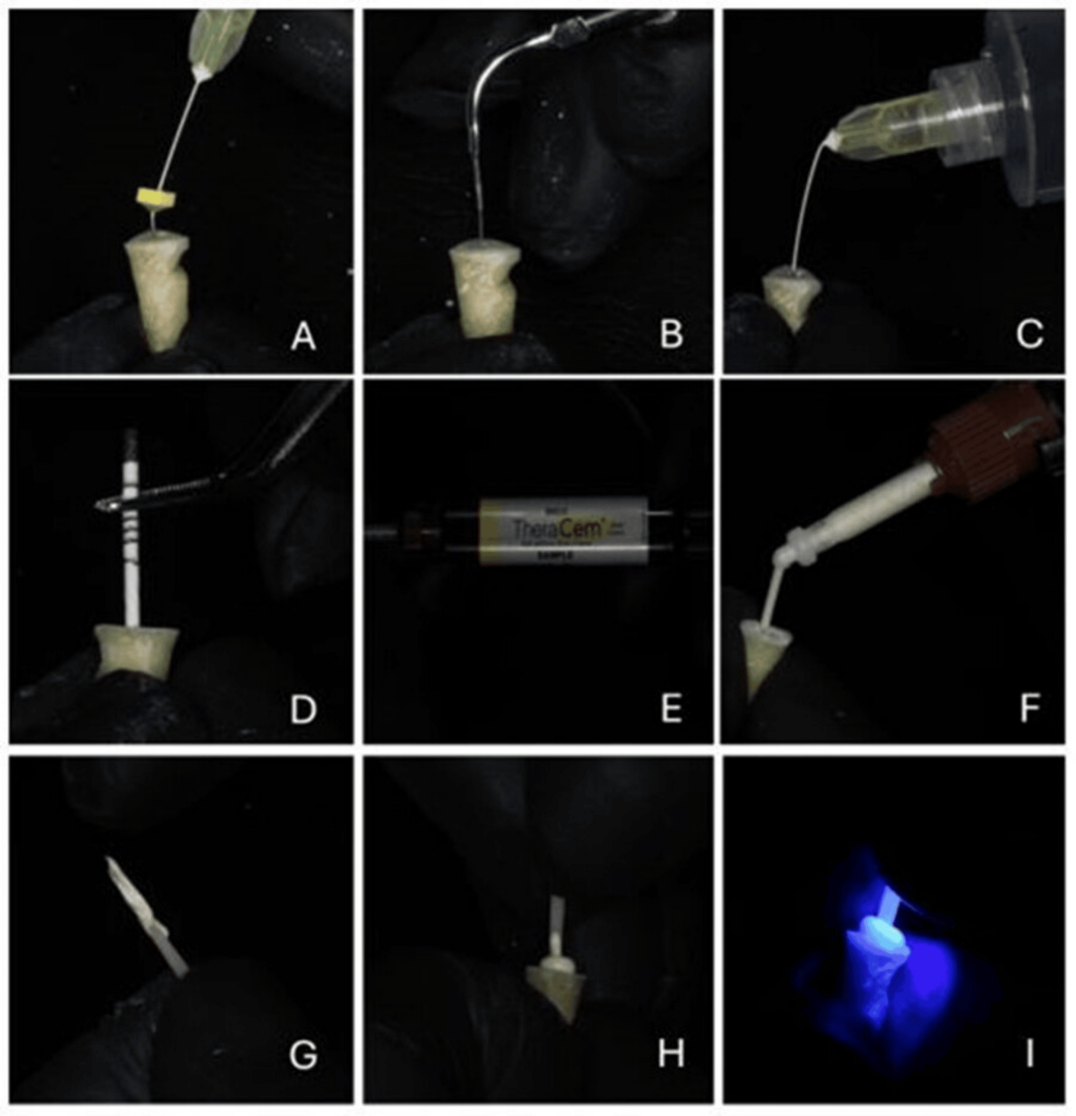
Cleaning and application of cement A, B, and C: Cleaning of the canal according to the intervention group; D: Removal of excess moisture; E: TheraCem; F: Application of cement (TheraCem, BISCO, Inc., Schaumburg, Illinois, USA); G, H, and I: Steps in cementing the post into the canal.

The samples were randomly divided into two groups, each containing seven teeth. Subsequently the post cementation protocol was carried out (Figure 1).

A radiographic verification of endodontic treatment was made, and the root length was measured via digital radiography using iView Rx software (Owandy Radiology, Croissy-Beaubourg, France; digital equipment from the Universidad del Valle). Next, the canal was unclogged with a low-speed drill and contra-angle handpiece, leaving an apical seal of at least 4 to 5 mm on the basis of the previously measured root length. The canal was then shaped the shaping drill of the DT Light Post^®^ Illusion^®^ X-RO^®^ fiberglass post system.

For group two (mechanical activation), the canal was cleaned with 10 ml of 5% NaoCl dispensed via a disposable hypodermic syringe with a 30 mm gauge needle (Monoject), and cleaning was performed with a low-speed rotational canal brush (Versa Brush™) activated for 10 seconds to chemically and mechanically clean the canal walls. The difference in activation times was due to the high frequency used by the ultrasound, which had to be spaced out to avoid overheating the tooth. Mechanical activation did not require as much time because of the low frequency of the vibrations from the rotating tips. The sample was then irrigated with 20 ml of sterile water to neutralize the NaOCl. 

For group one (ultrasonic activation), the canal was cleaned with 10 ml of 5% NaOCl dispensed via a disposable hypodermic syringe with a 30-gauge needle (Monoject), and a Biosonic S1 scaler (Coltene frequency of 28 kHz ± 3 kHz) with tip # 1 was placed in the canal to ultrasonically activate the NaOCl for 20 seconds to chemically and ultrasonically clean the canal walls. The sample was then irrigated with 20 ml of sterile water to neutralize the NaOCl. The excess moisture was removed with paper tips, which were applied until the tips were completely dry. Later, the post was cleaned with isopropyl alcohol and the cement (TheraCem, BISCO, Inc., Schaumburg, Illinois, USA) was injected into the canal with an intracanal tip while moving in the apical-to-coronal direction. Finally, the post was placed inside the canal and its seating was verified, and the sample was photoactivated for 30 seconds with an Ultradent Valo lamp at an intensity of 1200 mW/cm^2^.

All the samples were aged by storage in distilled water at a constant temperature of 36°C for 30 days (Hygrotherm, Bego, Germany). This procedure was used to assess the behavior of the resin materials since water is crucial for their deterioration and its effect is very pronounced when adhesive systems are used. Four cuts (each 2.5 mm thick) were made on each tooth with IsoMet^®^ 1000 Precision equipment (Buehler, Lake Bluff, Illinois, US) and a diamond disc (Isocut Wafering Blade-CBN HC, 7 inches in diameter (Buehler, Lake Bluff, Illinois, US)) under cooling conditions, yielding three root discs of the cervical, middle and apical zones, obtaining three samples per tooth, 21 per group, 42 samples in total.

To determine the adhesive strength between the cement and the root dentin at the interface, push-out tests were performed with a universal machine (Instron, Model 3366, Instron Corp., US). In this test, each sample was attached to the base of the universal machine with cyanoacrylate adhesive such that the coronal surface of the sample faced the machine. The dental disc was tested in the apical-to-cervical direction, and a cylindrical plunger (1 mm in diameter) was applied to the sample at a speed of 0.5 mm/min until the post became dislodged.

Additionally, a stereomicroscopy analysis (Stemi 2000C, Carl Zeiss, Germany) was performed on the samples to determine the type of failure after post removal. Two-factor ANOVA was performed to analyze differences among the zone and type of irrigation activation maximum load resistance (MPa) and determine the 95% confidence intervals and a significance level of 5%.

## Results

The results for maximum strength for group one (mean: 13.60±5.10 - median 13.09 (10.49-16.62)) are higher in comparison with group one (mean 11.62±4.13 - median 11.21 (9.46-13.29)), although no statistically significant differences were found (p: 0.1745). The ultrasound group showed greater resistance at maximum load, with an average resistance of 13.60 MPa. Similarly, analysis of the discs (cervical, middle, and apical) revealed that the average resistance to the maximum load for mechanical activation was greater in the apical third, with an average of 12.10 MPa, than in the ultrasound group, whose average resistance was 15 MPa in the apical third, without a statistically significant difference.

Considering the different discs generated in the ultrasonic activation group, the highest maximum resistance values were found in the apical cut. Similarly, the maximum resistance values were also detected in the apical section after mechanical activation (Table 1).

**Table 1:** Maximum resistance comparison

Cut	Maximum resistance (MPa)					
	Mechanical activation			Ultrasonic activation		
	Mean ± SD	Median (IQR)	p value	Mean ± SD	Median (IQR)	p value
Cervical	11.71±1.92	10.71(10.12-13.76)	0.8971	12.28±4.33	11.21(7.47-16.06)	0.6321
Medium	11.03±4.98	11.21(6.70-12.78)		13.51±7.35	12.81(8.08-21.52)	
Apical	12.10±5.25	11.56(9.46-14.24)		15.00±3.11	15.11(12.47-17.78)	

**Figure 2:**
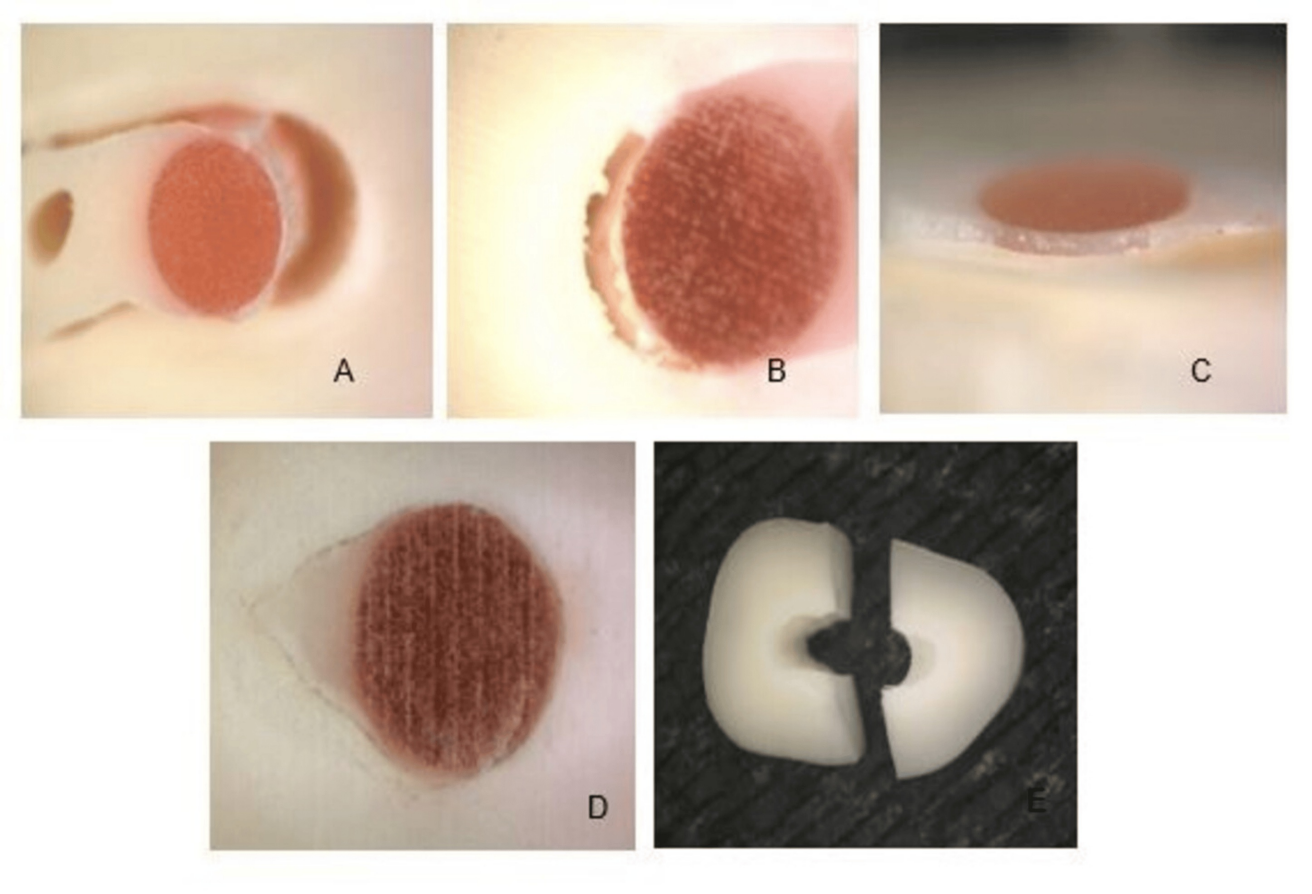
Types of faults A) Adhesive failure to dentin; B) Cohesive failure within cement; C) Adhesive failure to dentin; D) Cohesive failure within dentin; E) Dentine fracture.

Regarding the type of fault, adhesive to dentin (AD), adhesive to post (AP), cohesive to dentin (CD), and cohesive to post (CP) were detected. Although some cohesive failures were observed, both groups presented a higher prevalence of adhesive failure (Figure 2).

## Discussion

In this study, the adhesive strengths of fiberglass posts cemented in endodontically treated teeth with resinous cement (TheraCem) that had been irrigated with NaOCl activated in two different ways (mechanically and ultrasonically) were evaluated. Conventional activation of intracanal NaOCl did not eliminate organic tissue debris, microorganisms or detritus as desired. In 2024, Elfarraj et al. determined that the effectiveness of hypochlorite improved when sonic and ultrasonic devices were used for activation since the frequency of these devices allows access to the most difficult-to-reach places [6]. This result is consistent with the trends observed in this research, where group one presented maximum resistance values that were clearly higher than those in group two, possibly due to the activation system at the time of cleaning and likely because ultrasonic activation allows irrigation to penetrate well into the apical area, which is generally difficult to access.

In 2007, van der Sluis et al. reported that the concentration and ultrasonic activation duration of NaOCl are related to the complete removal of the dentin layer [7]. A study by Aveiro et al. in 2020 revealed that the synergy between NaOCl and ultrasonic activation increases the heat of the irrigation solution, which increases the dissolution of collagen and dentin tubule penetration [8]. This phenomenon was also observed in the present study, where the apical disc in the ultrasonic activation group presented the highest maximum load resistance value of 15 MPa.

After cementation with the self-adhesive cement, the apical cuts in groups one and two presented higher adhesive resistance values. This may have been influenced by the thickness of the cement, as the cement is thinnest in the apical cut, which allows greater contact between the post and the walls of the root dentin, as observed in the study by Kahnamouei et al. [14], who reported values of up to 16.72 MPa in the apical disc versus 4.38 MPa in the cervical disc.

In 2020, Mahrous et al. reported that TheraCem® exhibited high binding strength regardless of the substrate. The interaction between the resin cement and dentin creates a micromechanical penetration effect, which is considered the fundamental principle of its adhesion to the tooth substrate, in which the inorganic dental material is exchanged for synthetic resin [15]. In 2004, Goracci et al. [16] reported that push-out tests are safer and more effective than the microtensile technique for measuring adhesive strength, as push-out tests result in less sample loss, are easy to perform, and exhibit uniform distribution of the load at the adhesive interface [16-19].

The type of failure found is consistent with previously reported data with this type of mechanical test. In 2019, García et al. reported that adhesive failure predominated, with a 90.9% prevalence among the three cements evaluated [19]. The same result was observed in 2016 when Moreno-Preciado et al. evaluated adhesive resistance by means of eviction tests of fiberglass posts using different cementation protocols, where AD failure was 52.2% [18].

The main limitation of this study is the difference in the compressive strength of the bone compared to the artificial supports. However, due to the experimental nature of the study, this does not constitute a weakness.

## Conclusions

The ultrasonic activation group presented a higher average resistance at the maximum load than the mechanical activation group did, showing the advantages of ultrasound in terms of adhesive strength. Furthermore, with respect to the root thirds, we observed that the greatest resistance was in the apical third in both groups, although irrigation with ultrasonic activation may be more effective.

Despite the favorable results of ultrasonic activation, the study revealed no statistically significant differences. This indicates that, from a statistical perspective and within the limits of the study, both methods could be considered comparable in terms of adhesive strength. In cases where cleaning in the apical third is critical, ultrasonic activation may be preferred.
